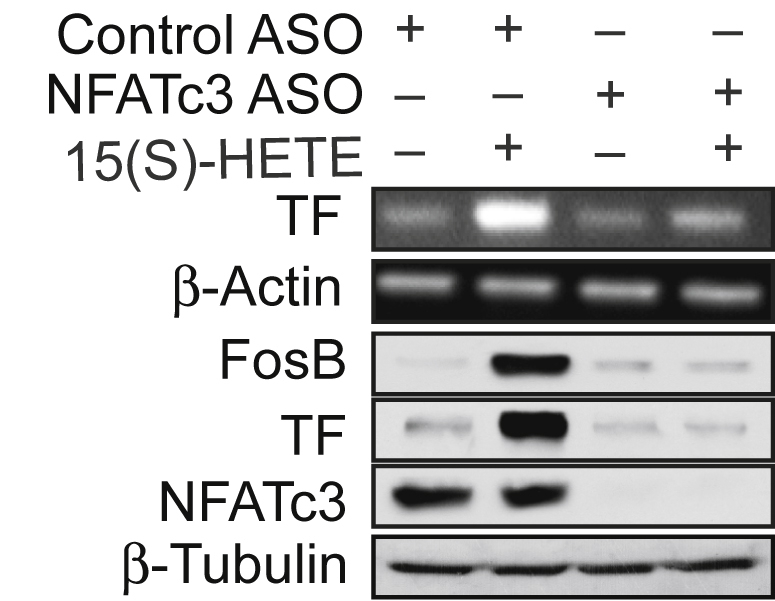# Correction: Heterodimers of the transcriptional factors NFATc3 and FosB mediate tissue factor expression for 15(*S*)-hydroxyeicosatetraenoic acid-induced monocyte trafficking

**DOI:** 10.1016/j.jbc.2022.101812

**Published:** 2022-03-15

**Authors:** Sivareddy Kotla, Nikhlesh K. Singh, Daniel Kirchhofer, Gadiparthi N. Rao

The β-tubulin blot in Figure 5B (middle panel) was inadvertently taken from Figure 5F (MEK1 blot in the third panel) while preparing the figures. The amended Figure 5B (middle panel) here shows the correct β-tubulin blot.

**Figure:**